# Intravesical Fungus Ball Caused by Candida albicans in a Diabetic Patient With Septic Shock: A Case Report

**DOI:** 10.7759/cureus.97790

**Published:** 2025-11-25

**Authors:** María Margarita Albarrán Gómez, Victor Manuel Molgado Garza, Carlos Manuel Cortés Becerra, Raquel González Garza, Juan Fermín Lozano Salinas, Adrian Gutiérrez González, Raquel Garza Guajardo, Rodolfo Franco Márquez, Adrian Camacho Ortiz, Jose Luis Maldonado Calderón

**Affiliations:** 1 Urology, Hospital Universitario Dr. José Eleuterio Gonzalez, Monterrey, MEX; 2 Infectious Disease, Hospital Universitario Dr. José Eleuterio Gonzalez, Monterrey, MEX; 3 Pathology, Hospital Universitario Dr. José Eleuterio Gonzalez, Monterrey, MEX

**Keywords:** bladder, candida infection, fungal bezoar, fungus ball, urinary tract infection, urosepsis

## Abstract

Fungal urinary tract infections (UTIs) are rare. In exceptional cases, fungal colonization can lead to the formation of a “Fungus ball” or fungal bezoar, which may cause urinary tract obstruction and sepsis. We report the case of a 61-year-old male with poorly controlled type 2 diabetes mellitus, presenting with septic shock and anuria. Imaging studies revealed an intravesical mass with air locules, and urine cultures grew *Candida albicans.* Despite systemic antifungal therapy, persistent obstruction required surgical management. A cystoscopy and open surgical approach allowed complete extraction of a fungal bezoar. Histopathological analysis confirmed fungal structures consistent with *Candida* spp. The patient recovered after systemic fluconazole therapy, with follow-up imaging showing resolution of obstruction. Urinary tract fungus balls are uncommon and often associated with predisposing factors such as diabetes mellitus, urinary stasis, and prolonged catheterization. Diagnosis requires a high index of suspicion supporting images. Treatment includes systemic antifungal therapy and, in selected cases, surgical removal. Although rare, a urinary fungus ball should be considered in diabetic patients with candiduria and obstructive findings on imaging. Early recognition and combined medical-surgical management are essential for favorable outcomes.

## Introduction

A fungus ball, also known as a fungoma or fungal bezoar, is defined as an aggregate of fungal hyphae adhered to cellular debris and urinary sediment [1]. It represents an uncommon complication of fungal urinary tract infections (UTIs), involving either the upper or lower urinary tract, and may result in urinary obstruction [1].

The prevalence of this entity remains unknown due to its rarity, with most available data derived from isolated case reports and small case series [1-7]. The most frequently implicated pathogen is *Candida albicans*, while reported risk factors include immunosuppression, poorly controlled diabetes mellitus, prolonged antibiotic therapy, long-term indwelling urinary catheters, urinary tract obstruction (either congenital or acquired), and female sex [1,8].

Clinical manifestations are often nonspecific and may include abdominal pain, lower urinary tract symptoms, urinary retention, and, in severe cases, septic shock. Therefore, a high index of clinical suspicion is required for diagnosis [1,9]. Imaging studies play a supportive role, as they can demonstrate mobile intravesical masses corresponding to aggregated fungal hyphae [2].

The management of fungal bezoars is not standardized and largely depends on the location and extent of the lesion. Therapeutic options include antifungal therapy (commonly amphotericin B or fluconazole), administered systemically, orally, or via bladder irrigation through urinary catheters. In selected cases, surgical intervention may be necessary for complete resolution. To date, no formal clinical guidelines exist to standardize management approaches [1,10].

We report the case of a diabetic patient with poor metabolic control who developed septic shock and obstructive uropathy secondary to an intravesical fungal bezoar as a complication of *Candida* infection, which ultimately required surgical removal for clinical resolution.

## Case presentation

A 61-year-old male with poorly controlled type 2 diabetes mellitus was admitted to the emergency department for altered mental status, fever, and anuria. On admission, his blood pressure was 80/60 mmHg, heart rate 123 bpm, respiratory rate 30 bpm, and body temperature 38°C. As physical examination revealed signs of urinary retention, a 16 Fr transurethral Foley catheter was placed to drain purulent urine (pyuria). 

Peripheral blood and urine cultures were obtained. Initial management included intravenous fluid resuscitation, followed by vasopressor support with norepinephrine and empiric antibiotic therapy with piperacillin-tazobactam, given the suspicion of urinary source sepsis.

Laboratory results showed a white blood cell count of 33.10 x 10^3^/µL, serum glucose of 617 mg/dL, and a serum creatinine of 3.8 mg/dL. Additionally, laboratory results are summarized in Tables 1, 2.

**Table 1:** Laboratory results at hospital admission. Laboratory findings revealed marked leukocytosis, severe hyperglycemia, and evidence of acute kidney injury.

Parameter	Result	Reference range
Complete blood count		
Hemoglobin	13.10 g/dL	12.20 - 18.10 g/dL
Hematocrit	42.70%	37.7 - 53.7%
White blood cells	33.10 K/µL	4.00 - 11.00 K/µL
Neutrophils	94.50%	37.0 - 80.0%
Platelets	298 K/µL	142.00 - 424.00 K/µL
Serum chemistry		
Glucose	617 mg/dL	60 - 100 mg/dL
Blood urea nitrogen (BUN)	69 mg/dL	7 - 20 mg/dL
Creatinine	3.8 mg/dL	0.6 - 1.4 mg/dL
Venous blood gas		
pH	7.34	7.32 - 7.43
pCO_2_	15 mmHg	40 - 45 mmHg
pO_2_	69 mmHg	---
Lactate	2.4 mmol/L	0.9 - 1.9 mmol/L
HCO_3_	8.1 mmol/L	24.0 - 30.0 mmol/L

**Table 2:** Urinalysis at admission. Urinalysis revealed pathological findings consistent with an infectious pattern.

Urinalysis parameter	Result	Reference range
Color	Reddish	---
Appearance	Very turbid	---
Specific gravity	1.01	1.005 - 1.025
pH	6	5.5 - 6.5
Proteins	152 md/dL	Negative
Glucose	Negative	Negative
Ketones	Trace	Negative
Blood	~200 RBCs/µL	Negative
Nitrites	Negative	Negative
Leukocyte esterase	~500 WBCs/µL	Negative
Yeast	Abundant with pseudohyphae	Absent
Bacteria	Abundant	Absent
Mucus	Absent	Absent

**Figure 1:**
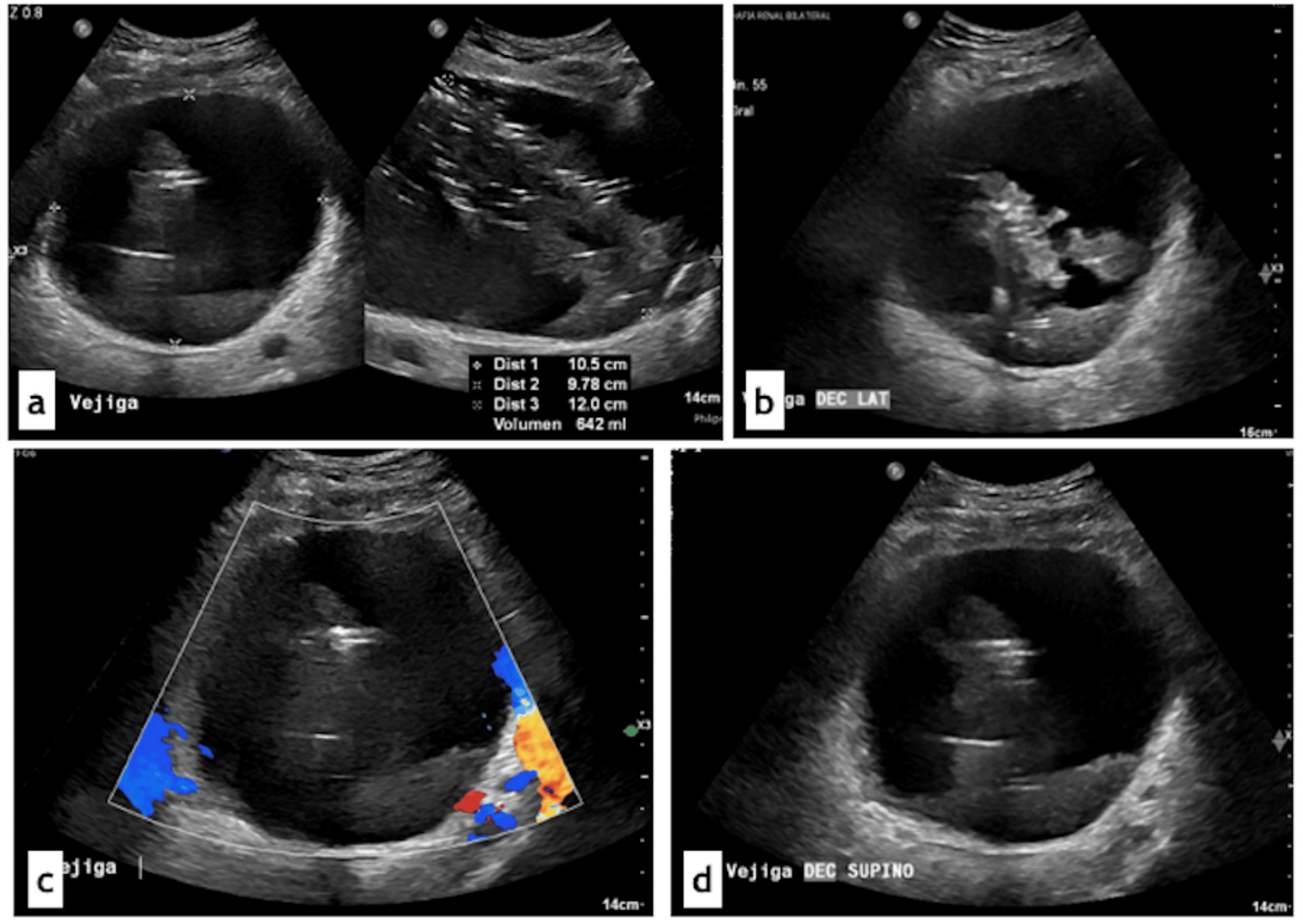
Bladder ultrasound. Bladder ultrasound demonstrated a bladder with an approximate volume of 642 cc. Inside, an irregular, mobile, hyperechoic features were identified, which changed position with patient movement. Multiple echogenic foci produced reverberation artifacts consistent with air locules. No ureteral jets were visualized. (a) Axial and sagittal bladder views showing an intravesical mass. (b) Axial view of the bladder depicting a mobile intravesical mass. (c) Image showing the absence of ureteral jets. (d) Axial bladder view showing sediment.

**Figure 2:**
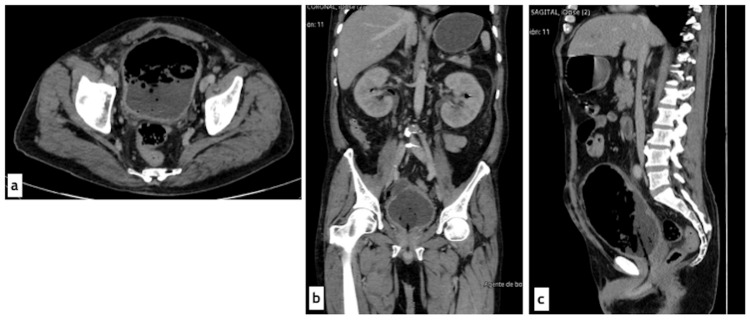
Abdominal computed tomography scan at admission. Contrast-enhanced abdominal computed tomography, venous phase: (a) Axial view at the pelvic cavity level showing a distended bladder with generalized wall thickening, intravesical sediment, and intravesical gas. (b) Coronal view at the renal level demonstrating emphysematous pyelonephritis (Huan-Tseng type IV), right-sided emphysematous pyelitis, bilateral ureteral dilation, and bladder findings similar to those described in (a). (c) Sagittal view showing marked bladder distention, urinary sediment, and abundant intravesical gas.

Imaging studies were requested, and bladder ultrasound demonstrated a distended bladder (642 cc) with an irregular, mobile intravesical mass with air locules (Figure 1). Abdominal computed tomography (CT) revealed acute emphysematous pyelonephritis (Huan-Tseng classification type IV), with bilateral dilation of the collecting systems and ureters along their entire course to the bladder, as well as findings consistent with emphysematous pyelitis and emphysematous cystitis (Figure 2).

The urinary catheter was replaced with a larger caliber Foley catheter (22 Fr), and broad-spectrum antibiotic coverage was continued. At 24 hours, the urine cultured yielded *Candida albicans*, and at 28 hours, peripheral blood cultures were also positive for *Candida albicans*. Consequently, intravenous fluconazole was initiated at a dose of 400 mg every 24 hours. 

**Figure 3:**
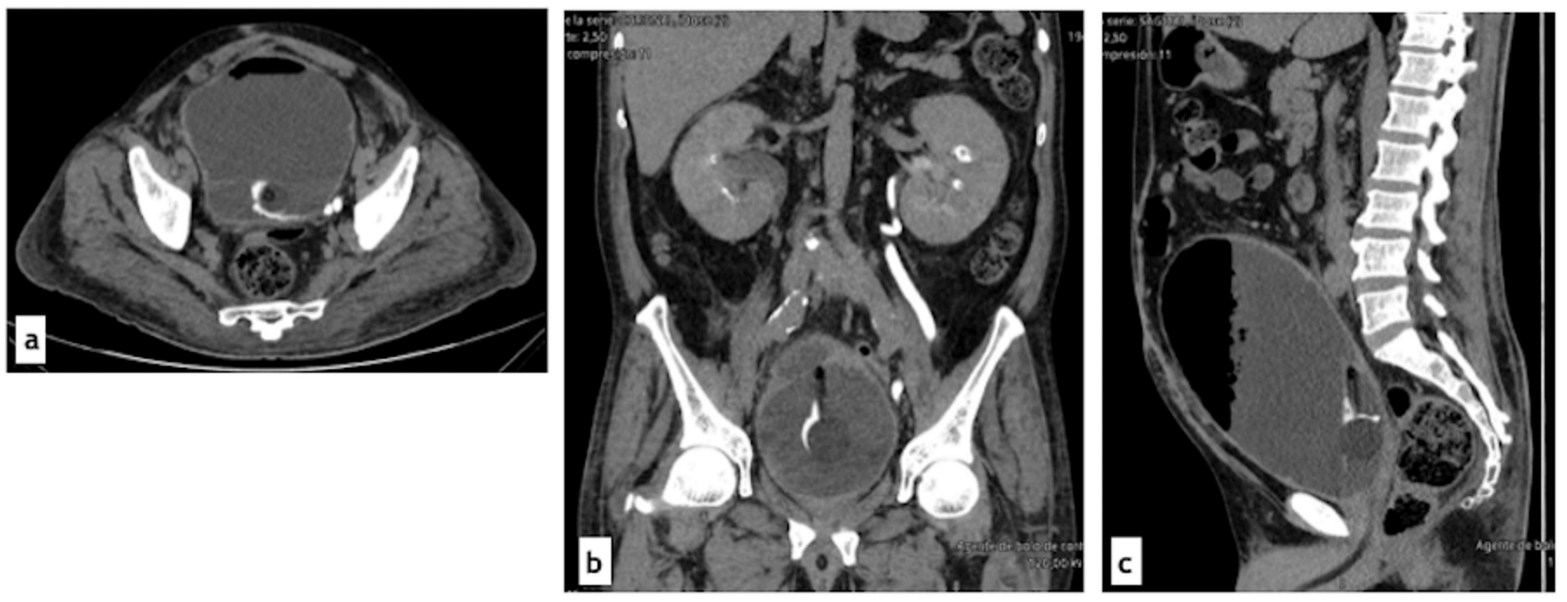
Follow-up abdominal computed tomography scan. Contrast-enhanced abdominal computed tomography, excretory phase: (a) Axial view showing persistence of intravesical sediment and gas; contrast medium delineates a crescent-shaped rim around the Foley catheter balloon without mixing with the sediment. (b) Coronal view demonstrating persistent bilateral ureteral dilation and bladder sediment non-enhancing with contrast medium. (c) Sagittal view revealing persistent bladder distention, urinary sediment, and intravesical gas.

The Foley catheter was maintained with persistent purulent output, and bladder irrigations with normal saline were started. However, due to recurrent catheter malfunction, multiple replacements were required. By the fifth day, vasopressor support was successfully withdrawn, but the patient remained febrile with worsening renal function (creatinine 7.4 mg/dL). A follow-up CT scan revealed persistent bilateral ureteral dilation and intravesical sediment (Figure 3).

Given these findings, a surgical approach was indicated. Cystoscopy using a 26 Fr sheath revealed areas of whitish fungal material and an amorphous fungal mass near the bladder neck. Partial endoscopic removal (approximately 30%) of the material was achieved; however, due to its gelatinous consistency and difficulty in extraction, the procedure was converted to open surgery.

**Figure 4:**
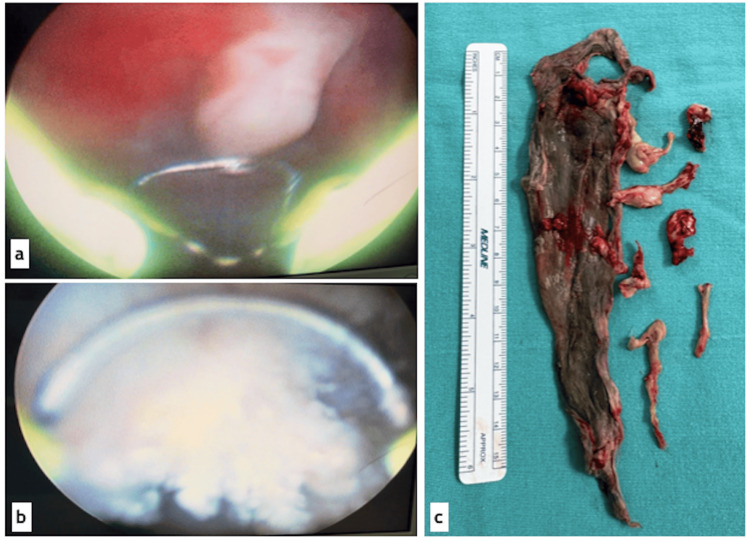
Intraoperative findings. (a) Cystoscopy showing bladder mucosal hyperemia and adherent whitish exudate. (b) Cystoscopic view of the mucosa covered with a thick, whitish plaque. (c) Specimen obtained from bladder exploration: brownish, soft tissue with irregular borders measuring 15 x 2 cm.

Through a cystostomy, the fungal ball was completely removed, followed by intravesical antifungal irrigation. Both ureteral orifices were catheterized with 5 Fr feeding tubes, and a cystostomy tube was placed through a counteropening, along with a transurethral Foley catheter. Continuous bladder irrigation was maintained at a low flow rate and discontinued on postoperative day two (Figure 4).

**Figure 5:**
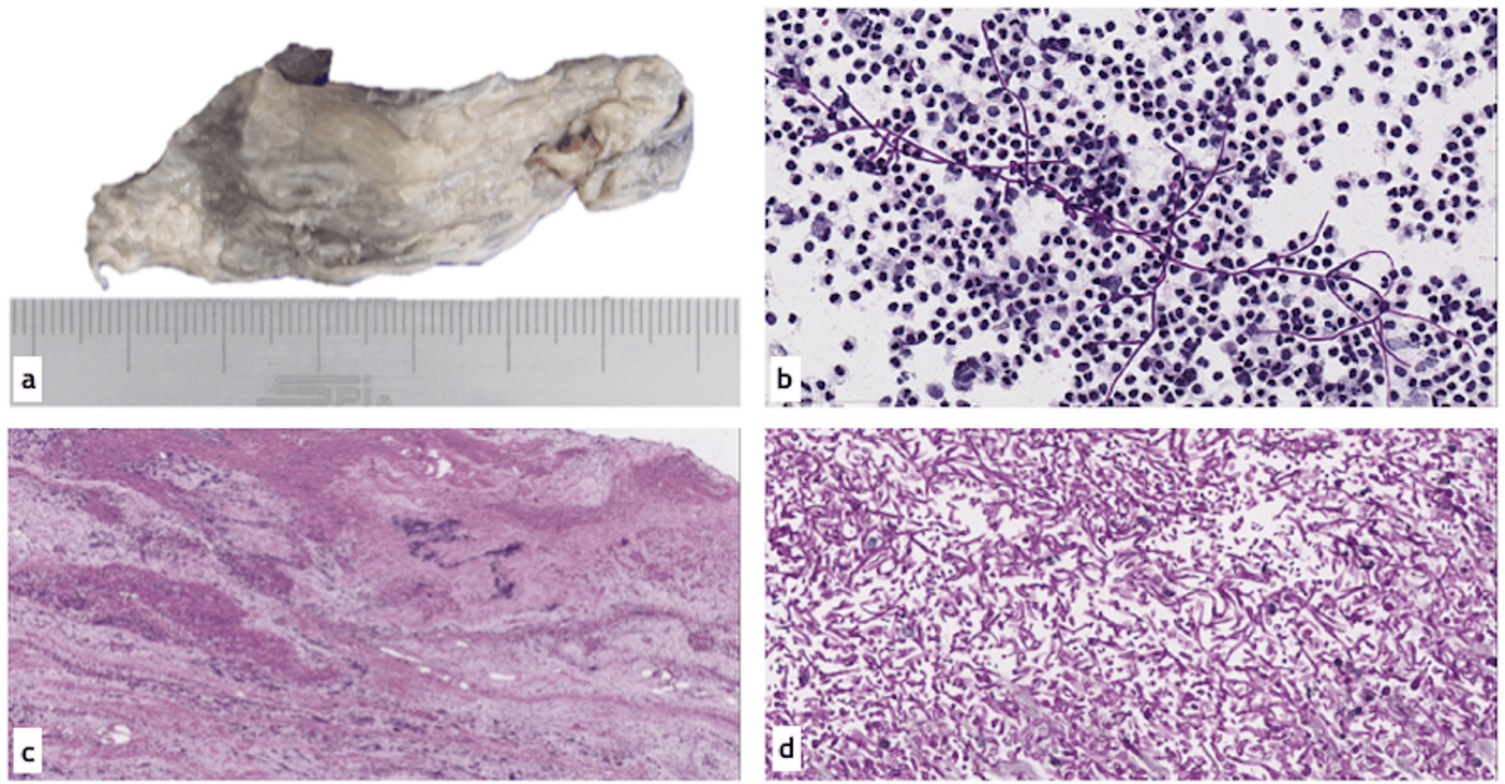
Histopathological findings. (a) Specimen corresponding to the bladder contents, consisting of a membranous tissue fragment measuring 7 x 2 cm, grayish in color with whitish areas, friable, and soft in consistency. (b) Spontaneous urine cytology, Papanicolaou stain, x400 magnification: showing pseudohyphae and yeast forms of microorganisms consistent with *Candida spp*., in a background of numerous polymorphonuclear leukocytes. (c) Panoramic view of the histological section of the specimen shown in (a), revealing amorphous eosinophilic tissue with calcifications. Hematoxyling and eosin stain, x40 magnification. (d) High-power view of the specimen in (a), showing numerous pseudohyphae and yeast forms as described above. Periodic acid-Schiff (PAS) stain, x400 magnification.

Samples were sent for histopathological examination, which reported bladder mucosa with necrosis and fibrinoleukocytic tissue associated with fungal structures morphologically compatible with *Candida spp*. The specimen identified as “bladder exploration producto” was diagnosed by the Pathology Department as a fungus ball (fungal benzoar). Additionally, periodic acid-Schiff (PAS) and Gomori-Grocott methenamine silver stains were performed, both yielding positive results for the presence of *Candida spp*. (Figure 5).

**Figure 6:**
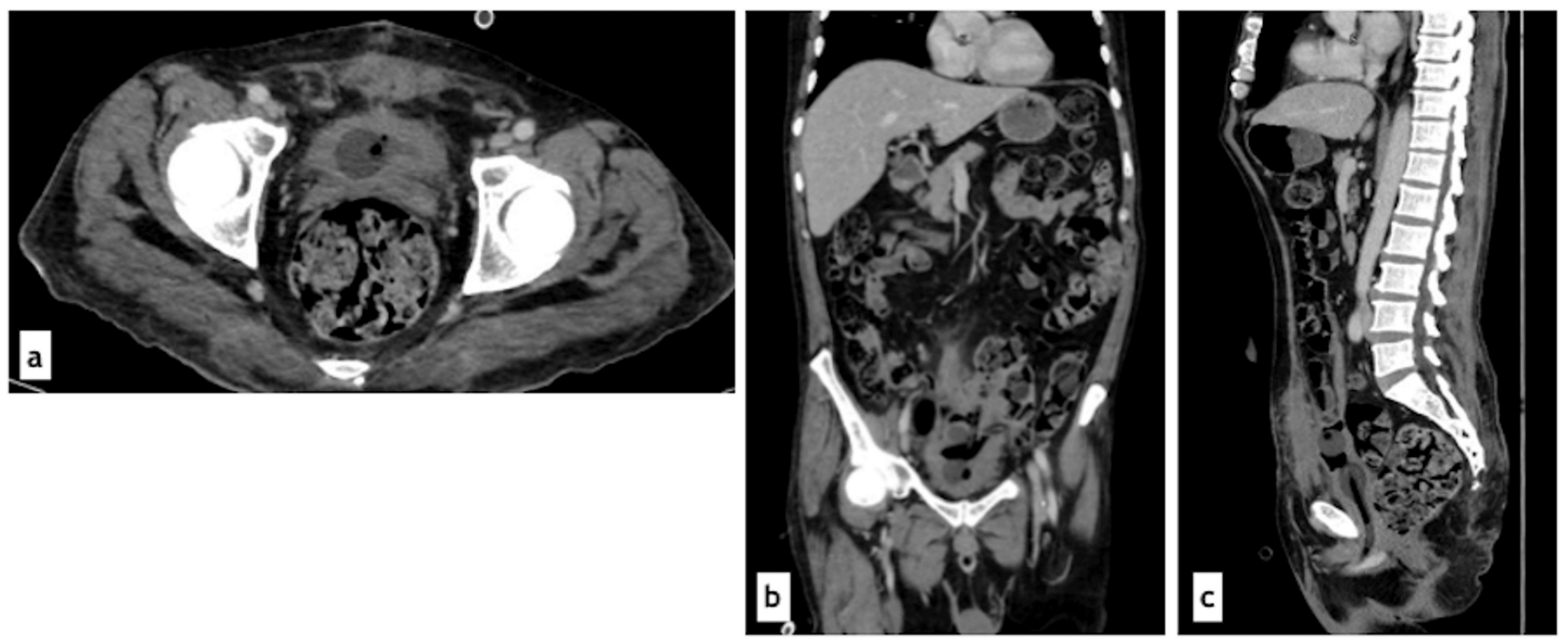
Postoperative abdominal computed tomography scan. Contrast-enhanced abdominal computed tomography, venous phase: (a) Axial view showing a partially collapsed bladder with a Foley catheter balloon inside. (b) Coronal view showing both urinary catheter balloons (cystostomy and transurethral) within the bladder. (c) Sagittal view demonstrating the same findings as (a) and (b).

Systemic antifungal therapy with fluconazole 800 mg/day was continued for 10 days after surgery. A follow-up CT scan demonstrated improvement of the nephrogram, resolution of the collecting system and ureteral dilation, and a bladder with both urinary catheters in adequate position, with no evidence of residual collections (Figure 6). Follow-up urine cultures were also performed, yielding negative results. The patient was discharged with both urinary catheters in place for urinary diversion and scheduled for outpatient follow-up.

## Discussion

Urinary tract infections (UTIs) are a common clinical condition, with bacterial etiology accounting for over 80% of cases and approximately 1% caused by fungal pathogens [1,11,12]. A fungus ball (also known as a fungoma or fungal benzoar) refers to an aggregation of fungal hyphae adhered to cellular debris and urinary sediment. It represents an uncommon complication of fungal infections of both the upper and lower urinary tract and can lead to urinary obstruction [1]. In about 95% of fungal UTIs, *Candida *species are isolated, with *Candida albicans* being the most frequently reported etiologic agent in cases of fungus ball as well [1,10,13]. The prevalence of urinary tract fungus ball remains unknown, as the condition is rare and most available evidence comes from isolated case reports or small case series [1-7].

The presence of *Candida* in the urinary tract is associated with several risk factors, including extremes of age, prolonged intensive care unit stay, chronic indwelling urinary catheters, spinal cord injury, renal transplantation, urinary tract obstruction (congenital or acquired), immunosuppression, female sex, broad-spectrum antibiotic use, and urinary tract instrumentation. Among diabetic patients, *Candida* growth in urine has been reported in up to 10% of samples [8,10,13].

The clinical presentation of a urinary fungus ball is often nonspecific, resembling bacterial UTIs [1]. Symptoms depend on the infection severity and can range from asymptomatic candiduria to severe urosepsis, including dysuria, stranguria, oliguria, polyuria, hematuria, pyuria, or pneumaturia (secondary to gas production), as well as abdominal pain, fever, chills, and general malaise [9].

Detection of yeasts in urinalysis presents a diagnostic challenge, as differentiation between contamination, colonization, or true infection requires careful interpretation [8,10]. Once *Candida* growth in urine is confirmed, it is essential to investigate predisposing anatomical or functional abnormalities that may facilitate fungal persistence [10]. Imaging studies play a key role in diagnosis, particularly when fungal cultures are positive [9]. Ultrasound is a practical and useful tool, as it can demonstrate mobile, heterogeneous, hypoechoic, and avascular intravesical masses on a Doppler examination [14]. On CT imaging, fungus balls may appear as intravesical gas or calcified masses. In non-contrast studies, these may be mistaken for urine; thus, excretory-phase CT is essential, as the lesions typically appear as filling defects or contrast-outlined rings within the bladder [14]. Additionally, imaging may reveal hydronephrosis secondary to urinary tract obstruction by fungal masses [1].

Management depends on severity and anatomical location (renal, ureteral, or bladder). Systemic fluconazole is the antifungal agent of choice due to its excellent activity against *Candida spp.*, while flucytosine and amphotericin B may be used as second- and third-line agents, respectively [10]. When the fungus ball is located in the kidney, nephrostomy drainage can facilitate urinary diversion and allow local irrigation with saline or amphotericin B, and in some cases, streptokinase instillation is performed to promote expulsion of the fungal mass [10,13,15]. For bladder or distal ureteral fungus balls, endourological extraction is the preferred approach [15]. Intravesical antifungal irrigation, usually with amphotericin B, has been reported; however, no standardized dosage or duration has been established [9].

In the present case, the main risk factor was poorly controlled diabetes mellitus. Additionally, diabetic cytopathy was suspected due to clinical findings of neuropathy, loss of lower limb sensitivity and hair, previous pressure ulcers, and absent bladder sensation despite significant distention. Diabetic neurogenic bladder dysfunction contributes to increased post-void residual volumes, promoting pathogen colonization and fungal growth [16].

Despite presenting with septic shock and identifiable risk factors at admission, the initial diagnosis did not consider a fungal UTI or bladder fungus ball, even though urinalysis showed abundant pseudohyphae and imaging revealed a mobile intravesical mass with bilateral hydronephrosis. Diagnosis was established after culture confirmation, worsening renal function, and persistent imaging abnormalities. 

According to the literature, fungus ball should be suspected in diabetic patients presenting with funguria, urinary obstruction, or abnormal bladder imaging findings, such as dense intravesical sediment, where cystoscopy is recommended for direct visualization [3,17,18]. In this case, imaging played a decisive role in diagnosis. Although findings are nonspecific, the mobile, avascular bladder mass on ultrasound, together with CT evidence of excessive urinary sediment and intravesical gas, were consistent with previously reported features [9,14].

The fungus ball was localized in the bladder, and endourological management was initially attempted; however, due to difficulty in complete extraction, conversion to open surgery was performed, as reported in other cases. Postoperative intravesical antifungal irrigation was not used, as its indication, dosage, and duration remain non-standardized [1,2,9,15]. Given the absence of standardized protocols, individualized multidisciplinary management remains essential.

## Conclusions

Diagnosis of a urinary tract fungus ball remains challenging due to its rarity, nonspecific clinical presentation, and limited description of imaging findings. Prompt recognition and management are critical, given the potential for rapid clinical deterioration. Systemic antifungal therapy is the cornerstone of treatment; however, in cases with persistent obstruction or lack of clinical improvement, surgical intervention becomes necessary.
